# Osteonecrosis of the Jaw and Concomitant Atypical Femoral Fractures with Bisphosphonates: A Comprehensive Literature Review

**DOI:** 10.7759/cureus.5113

**Published:** 2019-07-09

**Authors:** Veeraraghavan Meyyur Aravamudan, Chaozer Er

**Affiliations:** 1 Internal Medicine, Woodlands Health Campus, Singapore, SGP

**Keywords:** osteonecrosis of jaw, atypical femoral fractures, bisphosphonates

## Abstract

Bisphosphonates are the first line of treatment for osteoporosis. Complications of bisphosphonates, such as osteonecrosis of the jaw (ONJ) and atypical femoral fractures (AFF), have been reported in the medical literature. Concomitant occurrence of both of these complications is very rare. In this review article, we will discuss the etiology, pathogenesis, and clinical studies, as well as case studies and their management per the latest clinical guidelines.

## Introduction and background

Introduction

Osteoporosis is a common skeletal disease characterized by a reduction in bone strength and increased risk of fractures. Oral bisphosphonates are commonly prescribed for osteoporotic patients to arrest bone loss and preserve bone density. The safety of osteoporosis therapy is a matter of major importance for physicians who prescribe the drugs and patients who take them. Complications of bisphosphonates, such as osteonecrosis of the jaw (ONJ) and atypical femoral fractures (AFF), have been reported [1].In this review article, we will discuss the etiology, pathogenesis, and clinical studies on concomitant atypical femoral fractures and ONJ and their management per the latest clinical guidelines.

Materials and methods

We conducted a literature search of journal articles using the US National Library of Medicine PubMed database, PubMed, MEDLINE, Embase, Cochrane Library and Google Scholar databases, ClinicalTrials.gov for studies, and ISI Web of Science. No date restrictions were placed on the search. A thorough search for controlled clinical trials and cohort studies was done since the rarity of condition case reports were also included. We used the keywords "Bisphosphonates and atypical femoral fractures," "bisphosphonates and osteonecrosis of jaw," and "complications of Bisphosphonates."

Included studies were studies published in English that assessed the concomitant association of atypical femoral fractures and ONJ with bisphosphonates. Reference lists were also screened. From the search results, articles with irrelevant titles were discounted, with the remaining abstracts examined for relevance. The two authors of this review independently determined the eligibility of studies and assessed the methodology of the included studies. In this review article, we will discuss the etiology, pathogenesis, and clinical studies on concomitant atypical femoral fractures and ONJ and their management per the latest clinical guidelines.

Pathophysiology of atypical femoral fractures

Long-term bisphosphonates cause severe inhibition of osteoblasts and osteoclast, resulting in the suppression of bone turnover. At the same time, mineralisation of osteoid bone takes place, which causes the formation of brittle bone. This acellular brittle bone fails to remodel, resulting in microfractures [1].

Further, the formation of new blood vessels results in a reduction in vascular endothelial growth factor. In AFF, fractures at the subtrochanteric or diaphyseal region of the femur occur after minimal or no trauma.

Pathophysiology of ONJ

ONJ is characterised by infection, inflammation, bone resorption, and bone necrosis, but the sequence in which these occur has not been established. Bisphosphonate-related osteonecrosis of the jaw (BRONJ) is thought to be caused by trauma to dentoalveolar structures that have a limited capacity for bone healing due to the effects of bisphosphonate therapy. Other possible pathogenetic factors include suppression of bone turnover, altered immune status, and adverse effects of bisphosphonates on the oral mucosa [2].

Radiological appearance

Radiologically, the affected area shows thickening of the lateral femoral cortex and transverse fracture line.

## Review

Review of studies done on ONJ and AFF

This review showed that, although ONJ and AFF concomitant occurrence is rare, it can occur in patients receiving parenteral bisphosphonates (Table 1). ONJ can occur in AFF patients later on if they undergo dental procedures, it is worthwhile for the clinicians to look for this concomitant complication [1].

**Table 1:** Review of studies done on osteonecrosis of the jaw (ONJ) and atypical femoral fracture (AFF)

Study Author(s)	Title	Findings
Afif et al.	Do bisphosphonate-related atypical femoral fractures and osteonecrosis of the jaw affect the same group of patients? A pilot study.	Six ONJ patients were examined for signs of simultaneous occurrence of AFF and five AFF patients were examined for signs of simultaneous ONJ; there were no indications of simultaneous occurrence in any patients [1].
Behied et al.	Identification of risk factors for Bisphosphonate-associated atypical femoral fractures and osteonecrosis of the jaw in a pharmacovigilance database.	This case study found that there is no overlap between AFF and ONJ with all other bisphosphonate-related ADRs in terms of demographic variables, clinical characteristics, or concomitant drug treatments [3].
Graves et al.	Patients receiving parenteral bisphosphonates for malignant disease and having developed an atypical femoral fracture are at risk of concomitant osteonecrosis of the jaw: An evidence-based review.	This review found that 30% of AFF patients being treated with parenteral bisphosphonates were at risk of developing comorbid MRONJ [4].
Puhaindran et al.	Atypical subtrochanteric femoral fractures in patients with skeletal malignant involvement treated with intravenous bisphosphonates.	This study found that dose and duration of IV bisphosphonate treatments made no significant difference in the development of AFF in patients with skeletal malignancy [5].
Chang et al.	Atypical femur fractures among breast cancer and multiple myeloma patients receiving intravenous bisphosphonate therapy.	Results showed that of all patients receiving IV pamidronate with or without zoledronate for metastatic breast cancer and multiple myeloma, only six sustained an AFF; these patients received higher doses of IV bisphosphonates, had longer duration of treatment, and were more likely to have been exposed to zoledronic acid. Two of these patients went on to develop MRONJ [6].
Won et al.	Atypical femoral fracture combined with osteonecrosis of jaw during osteoporosis treatment with bisphosphonate.	This case report found that a 67-year-old female patient receiving an oral bisphosphonate during seven years for the treatment of osteoporosis with two year-long drug holidays developed BRONJ followed by AFF [7].
Kim et al.	Concurrent bisphosphonate-related bilateral atypical subtrochanteric fractures and osteonecrosis of the jaw on bone scintigraphy	This case study reported on an 82-year-old woman who had been taking bisphosphonates for seven years for the treatment of osteoporosis; she presented with bilateral thigh pain and a scan showed increased tracer uptake in her bilateral subtrochanteric femoral shafts and in the right mandible without evidence of metastatic bone disease. She was diagnosed with atypical subtrochanteric fractures and bisphosphonate-related ONJ [8].
Chiu et al.	Atypical femoral fractures shortly after osteonecrosis of the jaw in a postmenopausal woman taking alendronate for osteoporosis.	This case report of a 63-year-old woman with a 30-year history of rheumatoid arthritis three years of diabetes. She experienced spinal compression fractures at levels L3 and L4 and was prescribed alendronate 70 mg weekly for seven years; she developed ONJ and AFF within six months [9].
Payumo et al.	Osteonecrosis of the jaw and bilateral atypical femoral fracture both occurring during treatment for osteoporosis: A case report.	This case report described an 81-year-old, obese, diabetic, female admitted with persistent jaw pain after tooth extraction; she had a 14-year history of postmenopausal osteoporosis and was on intermittent, unsupervised treatment with alendronate, denosumab and ibandronate. The patient was noted with tenderness intraorally of tooth number 35 periapical region, and imaging revealed a sequestrum in the molar area of the left hemi-mandible. The patient was diagnosed with ONJ and had suffered transverse, non-comminuted sequential fractures at both proximal femoral shafts during the eighth and eleventh year of treatment with antiresorptive agents [10].
Pispati et al.	Oral bisphosphonate induced recurrent osteonecrosis of jaw with atypical femoral fracture and subsequent mandible fracture in the same patient: A case report.	This case report describes a 60-year-old patient who received oral bisphosphonate therapy for osteoporosis. She later developed ONJ, AFF, recurrent ONJ, and subsequent mandible fracture with delayed AFF union [11].
Sanchez & Blanco	Osteonecrosis of the jaw (ONJ) and atypical femoral fracture (AFF) in an osteoporotic patient chronically treated with bisphosphonates.	In this case study, a 87 year old Caucasian woman with osteoporosis who was taking alendronate suffered a fall and sustained a subtrochanteric fracture of the left femur; three years later she experienced loose teeth and had a molar removed and dentures made for her; however, the denture resulted in an ulceration in the gum of the mandible and a diagnosis of ONJ [12].

Patients on long-term bisphosphonate treatment are at higher risk of having complications from concomitant ONF and AFF [3]. Further, patients who are diagnosed with cancer (in particular multiple myeloma) [4] are at greater risk of complications from this concomitant diagnoses, and those with diabetes are at a higher risk of developing ONJ [13].

It is certainly possible for ONJ and AFF to simultaneously occur in the same patient during prolonged treatment with bisphosphonates and, as such, physicians should reconsider the use of bisphosphonates when they encounter these complications. If bisphosphonates are still prescribed, the patient should be counseled about these complications and monitored for the concomitant occurrence of complications from ONJ and AFF. In addition, ONJ can be detected on dental examination, although these examinations are not routinely performed by most physicians.

The presence of pain, swelling, and sequestrum on radiograph are strong indicators of an ONJ diagnosis. Surgical resection of necrotic bone, antibiotic therapy, and pain control were in congruence with the recommended stage-specific treatment strategies by the American Association of Oral and Maxillofacial Surgeons [14]. Hyperbaric oxygen as an adjunct treatment of ONJ has been shown to improve healing, but there is not enough evidence to recommend this treatment modality as a matter of course [14]. Discontinuation of antiresorptive therapy until soft tissue closure has occurred should also be considered, though there is limited data to support this.

Preventive measures against ONJ in patients on antiresorptive therapy involve maintenance of excellent oral hygiene and cessation of smoking. In addition, invasive dental procedures such as dental extractions or implants should be avoided if possible [10]. Teriparatide therapy can be considered as an alternative treatment for bisphosphonates [15].

For ONJ patients, radiographs of the side of the jaw opposite from the ONJ-affected area should be taken and checked routinely to look for an asymptomatic fracture. If bisphosphonates must be discontinued, ongoing metabolic management should still continue in the form of calcium and/or vitamin D supplements.

Limitations of this review include the limited number of clinical studies with small sample sizes and only a few isolated case reports. The demographics of patients are not homogeneous; a few reported a population with post-menopausal osteoporosis and others reported malignancy, which makes it difficult to pinpoint a cohort at a higher risk. More research is needed to really pinpoint the cohort at a higher risk but still, clinicians should carefully look for these complications when patients are on longterm bisphosphonates

## Conclusions

The association between bisphosphonates and atypical femoral fractures is complex. The duration of treatment seems to be directly related to the risk of atypical femoral fractures. The higher frequency of controlateral femur fractures suggests this could be a generalised process. Surveillance of patients with AFF is essential because bilaterality is a common feature and incipient stress fractures can happen in the controlateral limb. Radiographs of controlateral femur must be performed. A technetium or magnetic resonance imaging (MRI) scan should be considered. Conservative therapy and bed rest is advisable for minimal pain; teriparatide, reduced activity, and reduced weight-bearing should be implemented. When there is severe pain, prophylactic intramedullary nail fixation is advised.
